# Repeatability of cortisol stress response in the European sea bass (*Dicentrarchus labrax*) and transcription differences between individuals with divergent responses

**DOI:** 10.1038/srep34858

**Published:** 2016-10-05

**Authors:** A. Samaras, A. Dimitroglou, E. Sarropoulou, L. Papaharisis, L. Kottaras, M. Pavlidis

**Affiliations:** 1 Department of Biology, University of Crete, Heraklion, Crete, Greece; 2 Research and Development Department, Nireus Aquaculture S.A., Greece; 3 Institute of Marine Biology, Biotechnology and Aquaculture, Hellenic Centre for Marine Research, Heraklion, Greece

## Abstract

Understanding the stress responses of organisms is of importance in the performance and welfare of farmed animals, including fish. Especially fish in aquaculture commonly face stressors, and better knowledge of their responses may assist in proper husbandry and selection of breeding stocks. European sea bass (*Dicentrarchus labrax*), a species with high cortisol concentrations, is of major importance in this respect. The main objectives of the present study were to assess the repeatability and consistency of cortisol stress response and to identify differences in liver transcription profiles of European sea bass individuals, showing a consistent low (LR) or high (HR) cortisol response. The progeny of six full sib families was used, and sampled for plasma cortisol after an acute stress challenge once per month, for four consecutive months. Results suggest that cortisol responsiveness was a repeatable trait with LR and HR fish showing low or high resting, free and post-stress cortisol concentrations respectively. Finally, the liver transcription profiles of LR and HR fish showed some important differences, indicating differential hepatic regulation between these divergent phenotypes. These transcription differences were related to various metabolic and immunological processes, with 169 transcripts being transcribed exclusively in LR fish and 161 exclusively in HR fish.

Knowledge of the stress physiology of animals, and especially of the mechanisms regulating the physiological stress responses, are of importance in order to understand how animals respond to stressors and explain the biological significance of the intra-specific variation observed in these responses. This is also important for the welfare of farmed animals, since it could help in optimizing their husbandry and lead to a more appropriate selection of the breeding stock, especially in genetic selection breeding programs. In general, the effects of a stressor depend heavily on how an animal perceives and processes the stressful stimuli and how it can cope with it^1^. In this context, much attention has been given during the last decades to the fact that individuals of the same species may show consistently divergent physiological and behavioral responses to a stimuli or stressor. These sets of responses have been described as coping styles^1^, behavioral syndromes, personalities or temperaments, with a more or less synonymous use^2^. Two main categories of coping styles have been identified in vertebrates, namely the proactive and reactive^1^, that differ in many aspects of their behavior and physiology. In specific, proactive animals seem more aggressive, display the fight-flight response and show low behavioral flexibility, in contrast to reactive animals that tend to be non-aggressive and cautious, adopt the freezing response strategy and are behaviorally flexible. In terms of physiology, proactive animals have been linked to low stress axis output, regulated by low mRNA expression of CRH in the hypothalamus, and low glucocorticoid (cortisol or corticosterone depending on the taxa) production. Moreover, these animals show low parasympathetic and high sympathetic activity compared to reactive individuals which show the reverse pattern^3^. Therefore, identification of such intra-specific differences in cortisol responsiveness and better understanding of their impact in the animal performance and fitness would be beneficial towards better husbandry and selection of breeding stocks in genetic selection programs.

Divergence in the cortisol response has been described in many tetrapod vertebrates, including fish^2^,^4^, amphibians^5^, reptiles^4^, birds^6^, and mammals^7^,^8^. Fish, in particular, are among the most widely studied taxa in this aspect, and consistent differences in the cortisol response between individuals of the same species have been described^9^,^10^,^11^,^12^,^13^,^14^. Consequently, low (LR) and high (HR) responding fish have been identified in respect to the intensity and consistency of their cortisol response after exposure to acute stressors. Cortisol responsiveness has also shown moderate to high heritability in rainbow trout (h^2^ ranging between 0.22 to 0.56^15^,^16^,^17^,^18^), with strains of low and high responding individuals having been established^16^ though still merely used for research purposes. This divergence in cortisol responsiveness has also been associated with behavioral differences and coping styles^19^,^20^, as well as animal performance and fitness. Specifically, differences in growth^9^,^21^,^22^, plasma metabolites^12^,^23^, hepatic carbohydrates reserves and enzymatic activity^23^ and differential expression in many genes involved in physiological functions including metabolic and immune functions^24^ have been observed between LR and HR individuals.

Still however, individual divergence in cortisol responsiveness has not been identified in the European sea bass (*Dicentrarchus labrax*), a species of major economic importance in the Mediterranean aquaculture. This species shows an intense stress response^25^ and is considered susceptible to stress by inducing reproductive dysfunctions and disease outbreaks^26^, which, in turn, hamper production. Moreover, cortisol concentrations are characterized by high variability in both basal and response levels, so between studies^27^ as between individuals of the same population^28^. Consequently, it is of great importance to gain better knowledge on the stress physiology of this species by investigating the divergence of the response among individuals, since stress responsiveness can affect their performance^9^,^21^. Previous studies have shown low (h^2^ = 0.08^29^) to moderate (h^2^ = 0.34^30^) heritability of post-stress cortisol concentrations in this species, while LR and HR fish have not been identified. Yet, three suggestive Quantitative Trait Loci (QTL) for cortisol stress response have been identified^31^, indicating the existence of a genetic background regulating this response. It could be therefore suggested that an important confounding aspect in the studies showing low heritability^29^ or no consistency in the cortisol response^32^ in European sea bass might have been that the progeny used was produced from a breeding population with a small effective size and hence low genetic variability.

In this context, the main objectives of this study were to investigate the consistency of the cortisol stress response in European sea bass, to characterize possible differences in free cortisol concentrations among LR and HR individuals, and to get a better insight into the liver transcription profile of LR and HR fish. Such results could provide better knowledge of the stress physiology of the species, which could also constitute a potentially new selection trait to consider in selection programs for the species in aquaculture. Finally, analysis of the hepatic transcriptome between LR and HR fish could provide better insight to the metabolic and immune mechanisms that accompany the LR and HR phenotypes.

## Results

### Cortisol repeatability and identification of LR and HR fish

**Figure 1:**
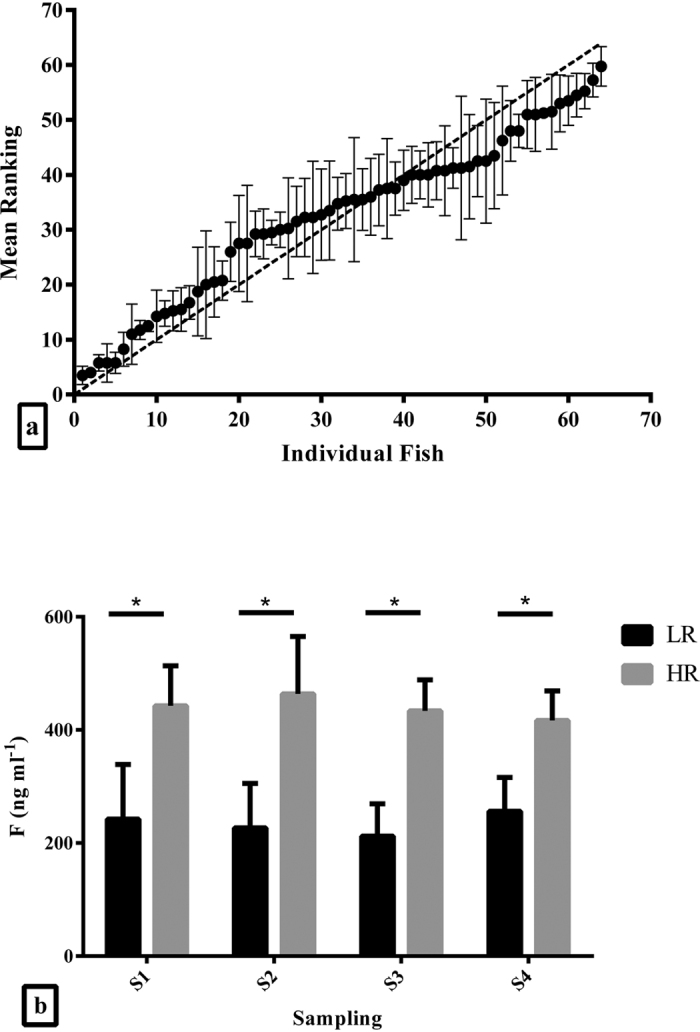
Cortisol concentration analysis. (**a**) Repeatability of ranked post-stress cortisol concentrations in E. sea bass (n = 64). Each point refers to the mean ranking of each fish ± S.E.M. The dotted line represents perfect repeatability, where one fish should have identical ranks across all trials. (**b**) Post-stress cortisol concentrations in all samplings for fish identified as LR and HR (n = 16 per group). An asterisk (*) indicates statistically significant differences between the two groups within each sampling point (S1 to S4) (*P* < 0.05).

Analysis of the post-stress plasma cortisol concentrations of the fish used in this study showed that this was a repeatable trait in European sea bass individuals, as estimated by the nested ANOVA (*r* = 0.389; F_58,129_ = 3.542; *P* < 0.001) (Fig. 1a). The measurement error of the repeated cortisol measures was estimated at 69.13 ng ml^−1^.

Moreover, based on the Z-scores analysis, it was possible to distinguish between fish that showed a consistently low or high acute stress cortisol response (Fig. 1). These two groups of fish (LR and HR; n = 16 per group) showed constantly different post-stress plasma cortisol concentrations in all samplings, with lower cortisol concentrations in LR than HR fish (F_1,30_ = 224.49; *P* < 0.001; Fig. 1b). The contribution of the 6 families to the LR and HR fish groups was unequal, while the family factor had a significant impact on cortisol responsiveness, explaining 28.54% of the total variance observed in this trait, as calculated by the variance components analysis.

Differences in resting cortisol concentrations were also observed between LR and HR fish sampled 15 days after the last exposure to the acute stress protocol. In particular, at the final sampling (S5), individuals identified as LR showed resting cortisol levels more than two times lower (93.4 ± 63.4, n = 16) than HR fish (203.3 ± 121.1, n = 16) (t_30_ = 3.216; *P* = 0.003).

A subsample of tested LR and HR fish (n = 10 per group) was also checked for the concentration of free cortisol in their plasma (Table 1). LR fish showed lower levels of both total (t_8_ = 7.48; *P* < 0.001) and free (t_8_ = 3.38; *P* = 0.01) cortisol concentration; however, the percentage of free over total cortisol (% Free) showed no difference between LR and HR fish (t_8_ = 0.26; *P* = 0.80; Table 1).

### Transcriptome analysis

**Figure 2:**
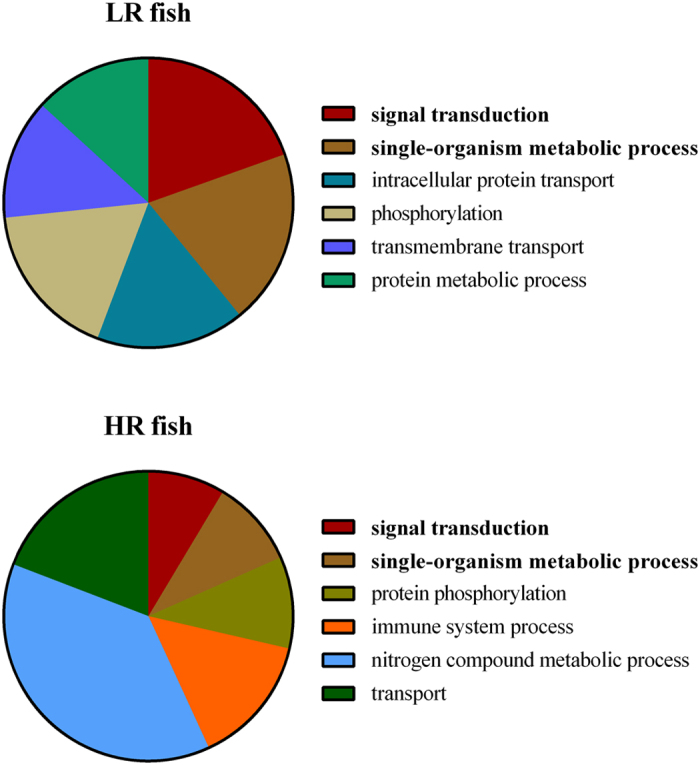
Multi-level profile of the Biological Processes Gene Ontology terms in (**a**) LR and (**b**) HR fish liver. GO terms with a node score below 5 were filtered out. Bold lettering indicates that these terms were shared between LR and HR fish.

The analysis showed that 169 transcripts were only transcribed in LR fish, and 161 transcripts were exclusively transcribed in HR fish. The transcripts were blasted against the European sea bass genome^33^ and categorized by the linkage groups they belonged to (Supplementary Table 1). Blast2Go analysis on the annotations of these transcripts showed that their putative functions included various metabolic processes, such as single-organism, protein, and nitrogen compound metabolic processes, as well as signal transduction (Fig. 2). In addition, they included several molecular functions, such as ATP binding, DNA, protein, metal ion and zinc ion binding, as well as transporter, transferase and protein kinase activity (Supplementary Figure 1). Unique metabolic processes observed only in LR individuals included protein metabolic processes, whereas immune system processes and nitrogen compound metabolic processes were only seen in HR fish, and consisted of more than 50% of the biological processes.

The Fisher’s exact test showed that 26 gene ontologies were significantly over-expressed in LR fish (Table 2) and 29 GOs in HR fish (Table 3).

**Figure 3:**
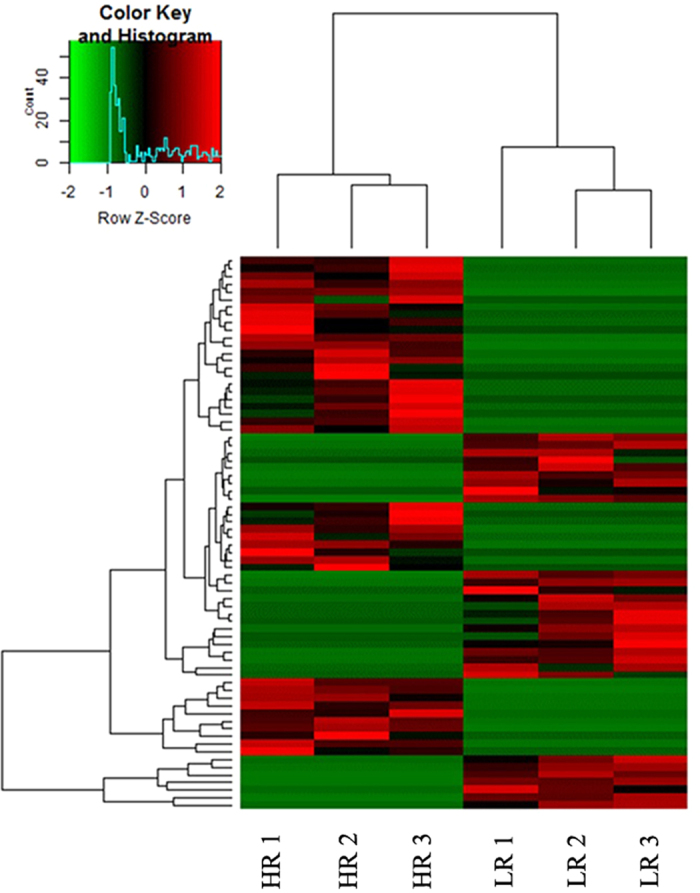
Heatmap of differentially expressed transcripts between HR and LR fish, as indicated under each column of clusters. Each row represents the expression of one transcript in relation to the other, while shades of red represent upregulation and shades of green represent downregulation.

Subsequently 72 transcripts related to the immune responses, carbon, protein, lipid, and energy metabolism that were differentially transcribed between LR and HR fish were selected and subjected to heatmap analysis (Fig. 3). Each row of the heat map represents the pattern of one transcript’s expression, while columns represent the HR and LR fish. This analysis grouped the LR and HR fish separately (Fig. 3).

## Discussion

The present study demonstrates for the first time the existence of individual-specific cortisol stress responses in European sea bass. Cortisol responsiveness was shown to be a repeatable trait, and fish showing constantly low or high responsiveness were identified and characterized as LR and HR fish. These fish also differed in their resting (*i.e.* without stress) cortisol levels, as well as the amount of free cortisol, which is the biologically active form of the hormone^34^. Finally, differences existed in the liver transcriptome profile between LR and HR fish, depicting differences in the regulation of metabolic and immune functions. Such results suggest that intra-specific differences should be taken into account when studying the stress responses in European sea bass, and could in turn explain the high variation observed in cortisol responsiveness. Moreover, knowledge of this aspect of stress physiology could assist in optimizing husbandry and welfare, as well as suggest cortisol responsiveness as a new potential selection criterion in genetic selection programs. It should, however, be kept in mind that selection for cortisol responsiveness could potentially correlate or be co-selected with other traits of interest such as growth^9^, and lysozyme response^9^,^15^, or not yet studied traits, which could exert positive, neutral or negative effects on fish performance.

LR and HR fish have been described in other fish species, such as rainbow trout^9^,^11^,^14^,^18^, Atlantic cod^10^, striped bass^13^, and gilthead sea bream^12^. However, up until today the cortisol response of European sea bass had been considered to show low to moderate heritability^29^,^30^. Additionally, it has not been feasible to identify LR and HR individuals^32^, possibly due to the low genetic variability of the population used, since fish were the progeny of few parents. Given the small number of families used in the present study, the heritability of this trait was not estimated, yet it was shown that the genetic background (*i.e.* family) had a significant impact on cortisol responsiveness. Whether the traits regulating resting cortisol as well as responsiveness to acute stressors are heritable and linked to specific QTLs in European sea bass or not is currently under investigation by our group.

Moreover, in the present study, differently responding fish seemed to differ in the resting concentrations of cortisol, with HR fish showing higher levels of circulating cortisol in their blood. To the best of our knowledge, there are only two studies showing direct differences in the resting (i.e. before stress) cortisol concentration between progeny of selected LR and HR families; in rainbow trout^35^ and in gilthead sea bream^36^. Significant positive correlations between family average post-stress and basal cortisol levels have also been shown between LR and HR families in rainbow trout^22^. However, prior to selective breeding towards the selection of divergent cortisol responses in rainbow trout, there were no differences between the basal cortisol levels of LR and HR fish^11^.

This is the first report on the concentration of free (*i.e.* unbound to proteins) cortisol in European sea bass. Free cortisol is the biologically active fraction of the hormone^34^. The concentration of free cortisol was higher in HR than LR fish, indicating that these fish indeed show higher levels of biological active cortisol and no regulatory mechanism takes place to diminish the differences observed in the total concentration. Nevertheless, even though the amount of free cortisol differed, the percentage of free in respect to the total concentration of cortisol showed no differences. Although in general the concentration of free cortisol was higher in the European sea bass when compared to others fish species, the percentage observed did not seem to differ from that in gadoids^10^,^37^, but was slightly higher than Atlantic salmon, *Salmo salar*^38^, and tiger pufferfish, *Takifugu rubripes*^39^. In line with the present study, no differences in the fraction of free cortisol were observed between LR and HR in Atlantic cod^10^. These differences in the free concentration of cortisol, both between species as well as within the same species between LR and HR fish, may reflect differences in the effects that cortisol could have on their performance, like immune function, metabolism and growth. It should however be noted that there are also other mechanisms regulating the actions of cortisol, like the abundance and affinity of the glucocorticoid receptors^40^.

Liver is an important organ that regulates many functions of the organism, including metabolism, immune functions and the stress-induced secondary responses^24^,^41^,^42^,^43^. In an attempt to associate the LR and HR phenotypes to the above-mentioned functions of the liver, a subset of LR and HR fish was subjected to hepatic transcriptome analysis. This analysis focused on transcripts that were exclusively transcribed in either LR or HR fish in order to identify the main crossing points between them. Specifically, 169 transcripts were transcribed only in LR fish, while 161 transcripts were exclusively transcribed in HR fish. These transcripts were annotated in metabolic processes that were shared between LR and HR fish, such as single-organism metabolic process (*i.e.* chemical reactions and pathways, including anabolism and catabolism, by which living organisms transform chemical substances and which involve a single organism) and signal transduction, and others like protein, and nitrogen compound metabolic processes that were included in LR or HR fish exclusively. In particular, intracellular protein transport, phosphorylation, transmembrane transport, and protein metabolic processes were unique to LR fish. On the other hand, nitrogen compound metabolic processes, transport, immune responses, and protein phosphorylation were unique to HR fish.

Out of all the biological processes exclusively annotated in the multi-level analysis in HR fish, nitrogen compound metabolic processes accounted for 37.7%. Moreover, enrichment analysis showed that glutaminase activity was significantly over-represented in HR fish, mediated by the transcription of *gls* transcript in HR fish only. The enzyme encoded by this transcript catalyzes the first reaction of the glutamate catabolism, and is therefore related to the metabolism of nitrogen and formation of glucose^44^. This enzyme is also involved in other amino acid metabolism, as shown by the KEGG pathways of alanine, aspartate and glutamate metabolism, as well as arginine and proline metabolism (Reference Pathway map00250 and map00330 respectively; KEGG PATHWAY Database; Kanehisa Laboratories; http://www.genome.jp/kegg/pathway.html). Increased cortisol concentrations have been shown to stimulate nitrogen metabolism in the sea raven, *Hemitripterus americanus*^45^, while nitrogen excretion tended to be unaffected by chronic cortisol stimulation in rainbow trout^43^.

Apart from metabolism, liver is also an important organ in the immune response of fish, synthesizing various immune-related proteins^46^. In this study, the GO term related to immune responses was annotated in the metabolic processes pie chart of HR fish (Fig. 2). In detail, the GO term of antigen processing and presentation of endogenous peptide antigen via MHC class I was enriched in HR fish, due to the high transcription of the *tapbp* transcript. Tapasin, a member of the MHC class Ia antigen-loading complexes, mediates the interaction between MHC class Ia molecules and the transporter associated with antigen presentation^47^. Apart from *tapbp*, other MHC class Ia related transcripts were transcribed in HR fish, like the *mhc class ia antigen* and *major histocompatibilty complex I-related gene*. Unlike the present results, in rainbow trout high levels of cortisol were related to a down-regulation of the MHC class II antigen beta chain and the MHC class II antigen associated invariant chain^41^. In LR fish, on the other hand, processes regarding the negative regulation of T-cell migration and cytokine production involved in inflammatory response were significantly enriched.

**Figure 4:**
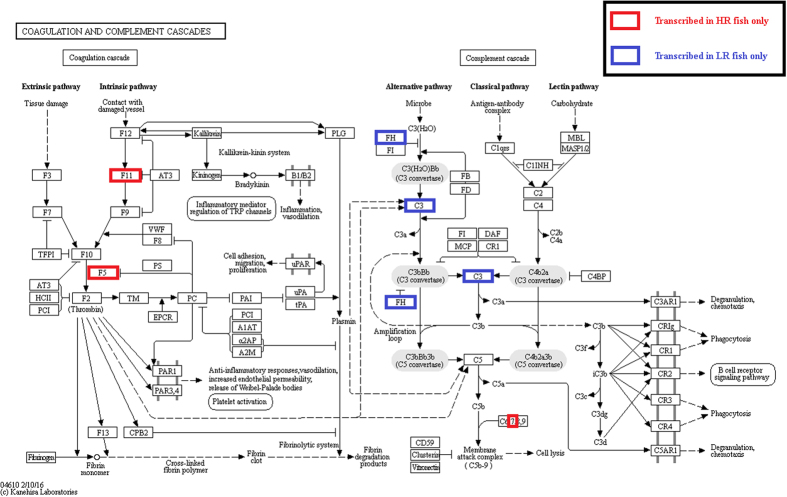
Annotated transcripts in the complement and coagulation cascades KEGG pathway. Blue and red boxes indicate annotated transcripts expressed in LR and HR fish exclusively.

Differences between LR and HR fish were also present in the expression of transcripts encoding for proteins involved in the pathway of complement and coagulation cascades (Fig. 4). Specifically, in LR fish, the liver transcripts encoding for complement C3 and Factor H (HF) proteins were transcribed when compared to HR fish (Fig. 4). C3 protein plays a central role in the activation of the complement system, which also forms the serine protease C3-convertase upon binding to factor B^48^. The H Factor, on the other hand, functions to accelerate the decay of the C3-convertase^48^. In HR fish, on the other hand, a different component of the complement system was transcribed, namely the *c7* (Fig. 4), which is an important component of the Membrane Attack Complex (MAC)^48^. Finally, transcription of transcripts related to the coagulation of blood was observed in HR fish; specifically, *f5* and *f11* (Fig. 4).

The above results indicate possible differences in the regulation of the immune functions between the LR and HR phenotypes. It is not, however, yet clear if these differences are of importance to the overall performance of the fish. Future disease resistance experiments could shed light in this aspect. Furthermore, although cortisol is known to affect the immune function of organisms, it is unclear whether cortisol *per se* is responsible for these differences, or selection for cortisol responsiveness is correlated with aspects of the immune system, such as lysozyme^9^,^15^ which is an important enzyme in the innate immune functions. In this context, although no data exists for the expression of these genes in LR and HR fish, when rainbow trout was faced with a chronic cortisol increase, via slow-releasing cortisol implants, a down-regulation in liver *c3* and *cfh* genes, as well as B factor (*Bf*) was observed^42^, which is in line with the current results that show no expression of these transcripts in the HR fish.

A series of carbohydrate metabolism-related transcripts were transcribed in HR fish. Processes mediated by the mitochondrial pyruvate dehydrogenase complex were significantly enriched in these fish as a result of the high transcription of the *pdk2*. This kinase phosphorylates the active form of the enzyme pyruvate dehydrogenase (PDH_a_) to its inactive form (PDH_b_), inhibiting in this way its activity^49^. This inhibition leads to the conservation of three-carbon compounds for gluconeogenesis, instead of metabolite flux through fatty acid and cholesterol synthesis, and the tricarboxylic acid cycle^50^.

In LR fish, on the other hand, the biosynthesis of coenzyme A process was significantly enriched. An excess of CoA in the cell can inhibit the activity of PDK in mammals, favoring in this way the oxidation of pyruvate through the TCA cycle for the production of energy, or fatty acids and cholesterol^51^. Additionally, *glut2*, which encodes for a protein that is likely responsible for glucose transport and uptake by the liver, was transcribed in LR fish^52^. In mammals, the expression of this gene increases under high glucose concentrations to enhance the insulin secretory response to glucose^52^. From all the above, it could be suggested that the conservation of energy and glucose synthesis in HR, in contrast to a tendency towards oxidation of pyruvate and uptake of glucose in the LR fish, could be indicative of increased energy demand or reduced appetite for food, given that all tanks were feed *ad libitum*, which may in turn affect the overall performance of the fish.

Processes involved in protein metabolism and modification showed some differential transcription between LR and HR fish. In general, cortisol is believed to exert proteolytic actions in fish^53^, and elevated transcripts of ubiquitin after acute stress have been observed in rainbow trout^53^. The ubiquitin-proteasome proteolytic pathway is of major importance in the degradation of proteins. In detail, different aspects of the ubiquitin-proteasome pathway were regulated in LR and HR fish. Specifically, in LR fish, protein autoubiquitination was significantly enriched while in HR fish the process of ubiquitin ligase protein binding was enriched. Additionally, in LR fish, the serine-type endopeptidase activity process, which is involved in protein degradation, was also enriched. The energy and amino acids released by this proteolysis are subsequently used in protein synthesis^54^. Transcripts related to protein modification processes were also enriched in HR fish, involving important processes like the regulation of protein phosphorylation and glycosylation as well as c-AMP dependent kinase regulation.

In conclusion, the present study showed for the first time that the cortisol stress response is a repeatable feature in European sea bass, and therefore LR and HR individuals can be identified. These fish differ in the concentration of both total and free post-stress as well as resting cortisol concentrations. Better understanding of these differences could be useful in genetic selection programs by introducing new potential selection criteria, given the important impacts that cortisol responsiveness may have on the performance and fitness of the fish. Specifically, these types of fish showed differences in their hepatic transcription profile, with transcripts being solely transcribed in LR or HR fish. Enrichment analysis of those transcripts, revealed many processes that were differentially expressed between these groups of fish. In short, nitrogen metabolism, and some aspects of the immune system were significantly more expressed in HR than LR fish, which highlights the need for further study of their ability to resist exposure to pathogens. HR fish also expressed transcripts related to the conservation of energy and glucose in contrast to LR fish that expressed transcripts involved in the flux of three-carbon molecules towards oxidation, suggesting in that way the possibility for a higher energy demand or reduced feed consumption by the HR fish, which could have important aquaculture applications.

## Materials and Methods

### Fish and housing conditions

Six full sib families of European sea bass were randomly chosen out of 96 families from the Nireus S.A. (Greece) family-based breeding program. All families were created on the same day from different parents (6 females and 6 males). Each of the 6 families consisted of 12 immature fish (17 months-old at the beginning of the experiment), randomly chosen from the whole progeny of each respective family (mean weigh (±SD) of 93.2 ± (20.5 g)). Each family was reared separately in open circulation tanks at Nireus S.A. research facilities. Fish were fed twice per day, for 6 days a week, using a commercial diet (Blue Line 45:20 3.5 mm, Feedus S.A., Greece). Throughout the experimental period, the photoperiod was set at 12L:12D, the water temperature was 18.20 ± 0.03 °C, and the salinity 27. Oxygen and pH ranged between 6–10 mg L^−1^ and 7.20–7.40, respectively.

### Sampling

In the first sampling, all fish per tank were confined by lowering the water of the tank and then chased with a net for 5 minutes. Fish were left confined for 30 minutes, and then carefully netted, immediately anaesthetized in ethylene glycol monophenyl ether (300 ppm; Merck; 807291; USA) and then weighed and tagged using unique glass PIT tags. Blood was immediately collected from the caudal vessel via heparanized syringes and centrifuged (2,000 g; 10 min), and the resulting plasma was stored at −20 °C until analyzed. The stress protocol was repeated once a month for four consecutive months, termed hereafter as S1 to S4.

Fifteen days following the completion of the last stress experiment (S4), a final sampling was performed to assess resting cortisol values and to collect liver samples for *mRNA* analysis (S5). Fish were immediately captured, euthanized with high doses of anaesthetic, and their blood was sampled as described previously. Liver samples collected for molecular genetic analysis were immediately frozen in liquid nitrogen and subsequently stored at −80 °C until analyzed.

### Z-score calculation, repeatability statistic, and LR and HR fish identification

In order to correct for variations in the cortisol data between the samplings the Z-score was used instead of the raw data. By calculating Z-scores of the individual post-stress cortisol levels for each sampling, the cortisol concentrations of each individual fish were standardized in respect to the overall mean and standard deviation of each respective sampling^14^.

The repeatability of the post-stress cortisol levels was assessed using the repeatability statistic, *r*^55^. The consistency of the response within an individual across the four samplings was tested by a nested ANOVA, with the factor Individual being nested in the factor Family. The null hypothesis was that individual Z-scores were inconsistent within individuals and hence the variable was not repeatable.

For the identification of LR and HR individuals, the sum of the Z-scores of all samplings was calculated for each individual fish. In this way, high responders should always have a positive Z-score, and therefore show a high sum of Z-scores. In contrast, low responders should always show lower cortisol concentrations than the overall mean, and have negative sum Z-scores. Finally, fish with either intermediate or no consistence response should have intermediate sum of Z-scores. In this manner, the Z-scores of all individuals were ranked, and fish belonging to the upper quartile of the distribution of Z-scores were identified as HR, and those belonging to the lower quartile as LR fish. This method ensures that both the intensity and the consistent of the cortisol response were taken into consideration when characterizing these types of responses.

### Analytical measurements

Plasma total and free (*i.e.* unbound) cortisol were measured by the use of a commercial enzyme immunoassay kit (DRG^®^ Cortisol ELISA, DRG^®^ International Inc, Germany). The performance of the kit has been previously evaluated with linearity and recovery tests in E. sea bass plasma samples^28^.

The separation of free and bound cortisol was performed by ultrafiltration of the plasma, as described by^37^. Briefly, 200 μl of plasma were loaded in centrifugal filter units (Centrifree^®^-MPS, Micropartition devices, Millipore Corp, USA) and centrifuged at 2,000 g for 30 min at 18 °C. Subsequently, the resulting filtrate with the free cortisol was stored at −20 °C until analyzed. Determination of free cortisol was performed in a subsample (n = 10) consisting of the LR and HR individuals with the lowest and highest sum of Z-scores.

### RNA extraction

Liver tissue from the 3 LR and 3 HR fish with the lowest and highest sum of Z-scores, respectively, was subjected to RNA extraction. Disruption of the samples was performed in liquid nitrogen using a mortar and pestle. After adding lysate buffer, the lysate was homogenized by passing it through a 20G needle attached to a sterile plastic syringe 5 times. RNA was subsequently extracted using the RNA extraction Kit II of Machinery Nagel (Dueren, Germany), according to the manufacturers’ instructions. RNA concentrations were determined using NanoDrop ND-1000 spectrophotometer (NanoDrop Technologies Inc., Wilmington USA) and the quality was assessed by gel electrophoresis as well as by RNA Nano Bioanalysis chip (Agilent 2100 Bioanalyzer).

### NGS Sequencing

Total RNA was submitted for Illumina 100 bp reads paired-end sequencing (RNA-Seq) to the Norwegian High Throughput Sequencing Centre. Tags reads were separated using a mulitplex identifier (MID). Evaluation of the reads was assessed using the freely available FastQC software program (version 0.10.0; http://www.bioinformatics.babraham.ac.uk/projects/fastqc). Low quality reads as well as adaptors were removed by Trimmomatic software^56^. Read assembly was obtained by applying Trinity, version 2012-06-08^57^. Raw sequence data as well as metadata were submitted to the Short Read Archive (SRA) database of NCBI available under the accession number SRP064240.

### Expression analysis

Paired end reads were mapped against the constructed reference transcriptome in order to assess expression abundances quantified by RSEM version 1.2.3^58^. Contigs with low read support were excluded from downstream analysis. Transcript expression profiles were assessed by the estimation of pairwise abundance of transcript between the two types of response using the R Bioconductor package DESeq^59^. The most stringent dataset of transcripts was used, by analyzing transcripts that were exclusively expressed in one condition (no transcripts identified in the other) and in all three replicates, with *p*-value < 0.05 and FDR-value < 0.05.

### Functional annotations and gene ontology

All transcripts obtained were submitted against the non-redundant protein database (nr) as well as the non-redundant nucleotide database (nr/nt) using the standalone BLAST tools (version 2.2.25); threshold cut-off e-values of 10^−6^ and 10^−10^, respectively, were chosen. Annotations and GO terms assignment were retrieved by Blast2Go software, while the reference pathway in Fig. 4 was obtained by KEGG database^60^.

### Statistical analysis

Statistical analysis was performed using the SigmaStat 3.1 statistical package (Systat Software, San Jose, CA, USA) and SPSS v.22 (IBM Corp., Armonk, NY, USA). Results are presented as means ± standard deviation. Two-way repeated measures analysis of Variance (ANOVA) tests were performed. The type of individual (LR vs HR) and sampling time (S1 to S4) were used as fixed factors, with their interaction being also checked, and the individuals as subjects. For the comparison of free cortisol in a subset of LR and HR samples, and of the resting cortisol levels (S5) between LR and HR individuals t-tests were used. In all statistical tests data were examined for normality using the Kolmogorov–Smirnov test and for homogeneity of variance using Levene’s test prior to analysis. Variance components analysis was performed using the VARCOMP command in SPSS.

### Ethical statement

All experiments were performed in accordance with relevant guidelines and regulations. Nireus S.A. research facilities are certified and have obtained the codes for the rearing and use of fish for scientific purposes (EL04-BIOexp-01). All procedures on fish used in this study were approved by the Departmental Animal Care Committee following the Three Rs principle, in accordance with Greek (PD 56/2013) and EU (Directive 63/2010) legislation on the care and use of experimental animals.

## Additional Information

**Accession code**: SRP064240.

**How to cite this article**: Samaras, A. *et al*. Repeatability of cortisol stress response in the European sea bass (*Dicentrarchus labrax*) and transcription differences between individuals with divergent responses. *Sci. Rep.* **6**, 34858; doi: 10.1038/srep34858 (2016).

## Supplementary Material

Supplementary Figure 1

Supplementary Table 1

## Figures and Tables

**Table 1:** Total, Free and % Free cortisol (F) in selected LR and HR fish from the S4 sampling (n = 10 per group).

	LR	HR
Total F (ng ml^−1^)	217.2 ± 58.6^*^	439.2 ± 31.1^**^
Free F (ng ml^−1^)	129.1 ± 43.9^*^	247.0 ± 85.1^**^
% Free	59.4 ± 13.4	61.8 ± 15.8

Asterisks indicate statistically significant differences between the two groups (*P* < 0.05).

**Table 2:** Gene Ontology terms significantly enriched in LR fish.

GO ID	GO Term	**p**-value	Genes
GO:0004030	aldehyde dehydrogenase [NAD(P)+] activity	1.49E-02	*aldh3a2*
GO:0051895	negative regulation of focal adhesion assembly	3.11E-02	*aldh3a2*
GO:0006081	cellular aldehyde metabolic process	3.27E-02	*aldh3a2*
GO:1900016	negative regulation of cytokine production involved in inflammatory response	2.63E-02	*apod*
GO:0071638	negative regulation of monocyte chemotactic protein-1 production	2.63E-02	*apod*
GO:0060588	negative regulation of lipoprotein lipid oxidation	2.63E-02	*apod*
GO:0002088	lens development in camera-type eye	2.63E-02	*apod*
GO:0010642	negative regulation of platelet-derived growth factor receptor signaling pathway	2.63E-02	*apod*
GO:2000098	negative regulation of smooth muscle cell-matrix adhesion	2.63E-02	*apod*
GO:0015485	cholesterol binding	2.79E-02	*apod*
GO:0042308	negative regulation of protein import into nucleus	3.27E-02	*apod*
GO:0048662	negative regulation of smooth muscle cell proliferation	4.40E-02	*apod*
GO:0000302	response to reactive oxygen species	4.83E-02	*apod*
GO:0030791	arsenite methyltransferase activity	1.49E-02	*as3mt*
GO:2000405	negative regulation of T cell migration	2.63E-02	*cdipt*
GO:0090344	negative regulation of cell aging	2.63E-02	*cdipt*
GO:0010842	retina layer formation	3.44E-02	*cdipt*
GO:0060219	camera-type eye photoreceptor cell differentiation	3.59E-02	*cdipt*
GO:0005758	mitochondrial intermembrane space	1.49E-02	*cycs*
GO:0004714	transmembrane receptor protein tyrosine kinase activity	5.70E-03	*fgfr1a*
GO:0090140	regulation of mitochondrial fission	2.63E-02	*march5*
GO:0051865	protein autoubiquitination	4.40E-02	*march5*
GO:0042981	regulation of apoptotic process	3.59E-02	*nod1*
GO:0004594	pantothenate kinase activity	1.98E-02	*pank1*
GO:0015937	coenzyme A biosynthetic process	2.47E-02	*pank1*
GO:0004252	serine-type endopeptidase activity	3.06E-02	*tmprss6*

**Table 3:** Gene Ontology terms significantly enriched in HR fish.

GO ID	GO Term	*p*-value	Genes
GO:0003993	acid phosphatase activity	2.22E-02	*acp5*
GO:0000276	mitochondrial proton-transporting ATP synthase complex, coupling factor F(o)	2.95E-02	*atp5a1*
GO:0004089	carbonate dehydratase activity	2.22E-04	*ca5a*
GO:0006730	one-carbon metabolic process	3.49E-03	*ca5a*
GO:2000021	regulation of ion homeostasis	1.86E-02	*ca5a*
GO:0009264	deoxyribonucleotide catabolic process	1.12E-02	*dera*
GO:0004139	deoxyribose-phosphate aldolase activity	1.12E-02	*dera*
GO:0004359	glutaminase activity	1.49E-02	*gls*
GO:0008375	acetylglucosaminyltransferase activity	7.17E-03	*mgat1*
GO:0006486	protein glycosylation	4.28E-02	*mgat1*
GO:0005967	mitochondrial pyruvate dehydrogenase complex	7.46E-03	*pdk2*
GO:0006111	regulation of gluconeogenesis	7.46E-03	*pdk2*
GO:0010510	regulation of acetyl-CoA biosynthetic process from pyruvate	7.46E-03	*pdk2*
GO:0004740	pyruvate dehydrogenase (acetyl-transferring) kinase activity	7.46E-03	*pdk2*
GO:0005739	mitochondrion	1.16E-02	*pdk2 ca5a atp5a1*
GO:0072332	intrinsic apoptotic signaling pathway by p53 class mediator	1.49E-02	*pdk2*
GO:0008286	insulin receptor signaling pathway	2.04E-02	*pdk2*
GO:0042593	glucose homeostasis	2.22E-02	*pdk2*
GO:0031670	cellular response to nutrient	2.22E-02	*pdk2*
GO:0034614	cellular response to reactive oxygen species	2.41E-02	*pdk2*
GO:1902494	catalytic complex	2.77E-02	*pdk2 prkar1a*
GO:0006885	regulation of pH	3.32E-02	*pdk2*
GO:0005952	cAMP-dependent protein kinase complex	3.52E-04	*prkar1a*
GO:0008603	cAMP-dependent protein kinase regulator activity	4.54E-04	*prkar1a*
GO:0001932	regulation of protein phosphorylation	3.72E-02	*prkar1a*
GO:0019885	antigen processing and presentation of endogenous peptide antigen via MHC class I	7.46E-03	*tapbp*
GO:0031625	ubiquitin protein ligase binding	4.40E-02	*ube2w*
GO:0000139	golgi membrane	4.68E-02	*ube2w*
GO:0071218	cellular response to misfolded protein	7.46E-03	*ube2w*
