# Supplementary material for: Repeatability of cortisol stress response in the European sea bass (Dicentrarchus labrax) and transcription differences between individuals with divergent responses
Source: Sci Rep. 2016 Oct 5;6:34858. doi: 10.1038/srep34858 (PMC5050510; doi:10.1038/srep34858)
Supplement: Supplementary Figure 1 [file srep34858-s1.pdf]

Title

Repeatability of cortisol stress response in the European sea bass (*Dicentrarchus labrax*) and transcription differences between individuals with divergent responses

Authors

A. Samaras[*1], A. Dimitroglou[2], E. Sarropoulou[3], L. Papaharisis[2], L. Kottaras[2], M. Pavlidis[1]

Affiliations

1. Department of Biology, University of Crete, Heraklion, Crete, Greece
2. Research and Development Department, Nireus Aquaculture S.A., Greece
3. Institute of Marine Biology, Biotechnology and Aquaculture, Hellenic Centre for Marine Research, Heraklion, Greece

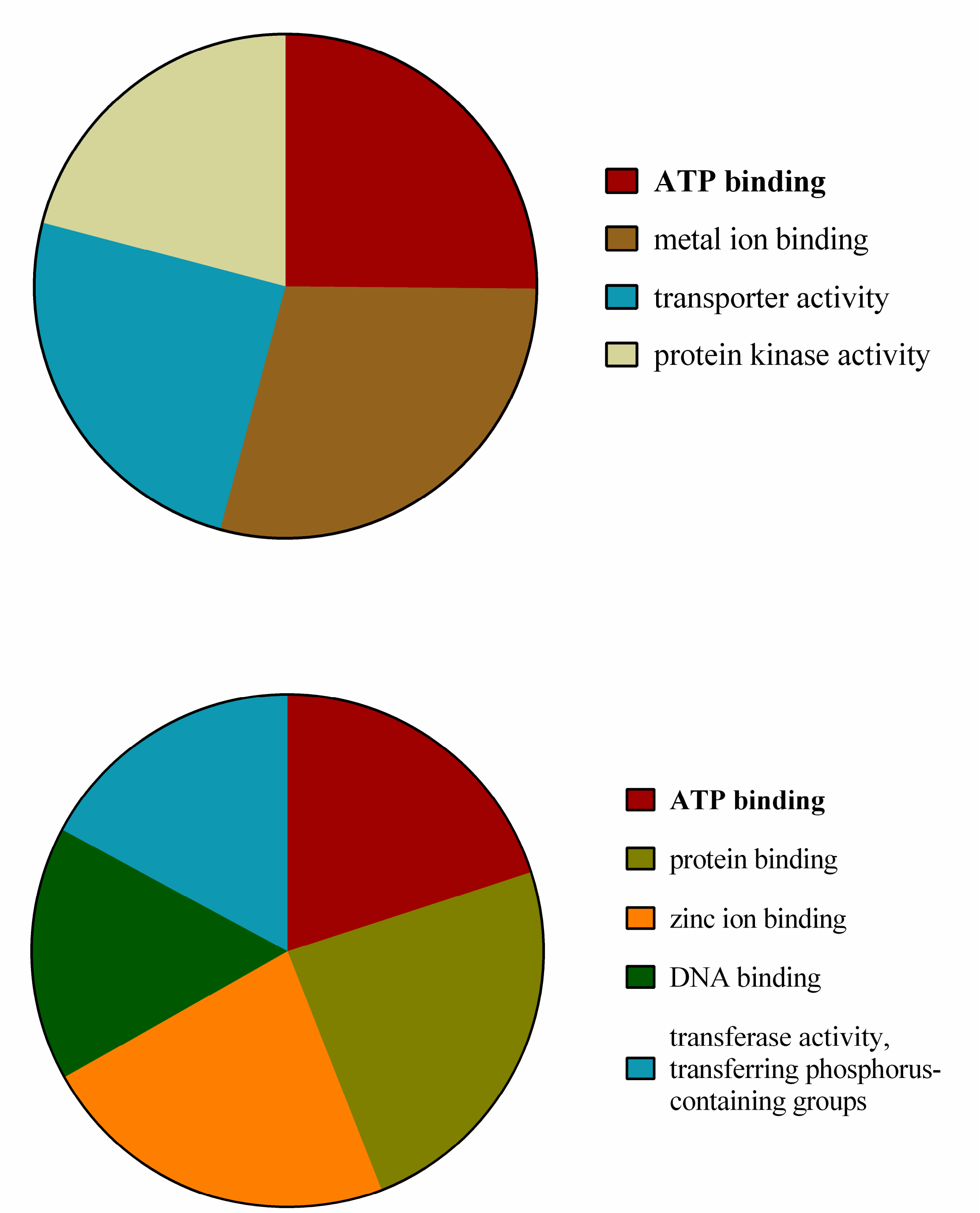

Supplementary Fig. 1. Multi-level profile of the Molecular Functions Gene Ontology terms in (a) LR and (b) HR fish liver. GO terms with a node score below 5 were filtered out. Bold lettering indicates that these terms were shared between LR and HR fish.
