# Supplementary material for: Repeatability of cortisol stress response in the European sea bass (Dicentrarchus labrax) and transcription differences between individuals with divergent responses
Source: Sci Rep. 2016 Oct 5;6:34858. doi: 10.1038/srep34858 (PMC5050510; doi:10.1038/srep34858)
Supplement: Supplementary Table 1 [file srep34858-s2.pdf]

Title

Repeatability of cortisol stress response in the European sea bass (*Dicentrarchus labrax*) and transcription differences between individuals with divergent responses

Authors

A. Samaras[*1], A. Dimitroglou[2], E. Sarropoulou[3], L. Papaharisis[2], L. Kottaras[2], M. Pavlidis[1]

Affiliations

1. Department of Biology, University of Crete, Heraklion, Crete, Greece
2. Research and Development Department, Nireus Aquaculture S.A., Greece
3. Institute of Marine Biology, Biotechnology and Aquaculture, Hellenic Centre for Marine Research, Heraklion, Greece

Supplementary Table 1. Transcript distribution in European sea bass linkage groups for the transcripts exclusively expressed in LR or HR fish. Sequences were blasted against the species genome, published by Tine et al. (2014) [33]. Only the transcripts that were successfully attributed to a linkage group are shown.

LR Fish	
**Transcript**	**Linkage Group**
comp24049_c0_seq5	LG1A
comp24202_c0_seq7	LG1A
comp24488_c1_seq5	LG1B
comp13351_c0_seq1	LG1B
comp20086_c0_seq2	LG1B
comp24371_c1_seq15	LG1B
comp24231_c0_seq7	LG2
comp24945_c2_seq1	LG3
comp10172_c0_seq2	LG3
comp18829_c1_seq2	LG3
comp24410_c0_seq6	LG4
comp24001_c0_seq29	LG4
comp20055_c0_seq4	LG4
comp24001_c0_seq40	LG4
comp24699_c1_seq7	LG4
comp20055_c0_seq3	LG4
comp21376_c0_seq1	LG4
comp16083_c0_seq1	LG4
comp5422_c0_seq1	LG4
comp20676_c0_seq2	LG5
comp14772_c0_seq1	LG5
comp24967_c1_seq37	LG5
comp22390_c0_seq3	LG5
comp21833_c0_seq5	LG5
comp24594_c0_seq2	LG6
comp16666_c0_seq2	LG6
comp23448_c0_seq3	LG6
comp19220_c0_seq1	LG6
comp22982_c1_seq2	LG6
comp24544_c0_seq20	LG7
comp17949_c0_seq2	LG7
comp24894_c0_seq38	LG7
comp23347_c1_seq2	LG7
comp24557_c0_seq8	LG7
comp24894_c0_seq45	LG7
comp24642_c0_seq13	LG7
comp14917_c0_seq1	LG7
comp24440_c0_seq5	LG8
comp22725_c0_seq10	LG8
comp15895_c0_seq1	LG8
comp22625_c0_seq1	LG8

HR Fish	
**Transcript**	**Linkage Group**
comp24886_c2_seq11	LG1A
comp24049_c0_seq2	LG1A
comp21881_c0_seq1	LG1A
comp23083_c1_seq9	LG1B
comp24897_c1_seq1	LG2
comp19685_c0_seq3	LG2
comp22406_c0_seq1	LG2
comp24738_c0_seq19	LG2
comp24777_c0_seq9	LG3
comp25017_c0_seq18	LG4
comp23737_c4_seq10	LG4
comp4520_c0_seq1	LG4
comp20676_c0_seq1	LG5
comp24386_c1_seq26	LG5
comp17232_c0_seq1	LG5
comp22726_c1_seq3	LG5
comp24594_c0_seq7	LG6
comp18318_c0_seq2	LG6
comp22813_c1_seq1	LG6
comp21938_c0_seq2	LG6
comp25012_c0_seq27	LG6
comp23203_c0_seq1	LG6
comp19220_c0_seq2	LG6
comp22813_c1_seq4	LG6
comp20860_c0_seq2	LG6
comp24963_c0_seq110	LG7
comp23193_c0_seq5	LG7
comp23531_c0_seq1	LG7
comp5425_c0_seq2	LG7
comp24507_c1_seq3	LG7
comp10076_c0_seq2	LG8
comp20447_c0_seq5	LG8
comp20447_c0_seq2	LG8
comp19847_c1_seq2	LG8
comp24103_c0_seq3	LG8
comp22973_c0_seq2	LG9
comp24046_c0_seq3	LG9
comp21377_c0_seq5	LG9
comp22059_c0_seq2	LG9
comp24769_c0_seq33	LG9
comp22701_c0_seq5	LG9

comp22973_c0_seq1	LG9
comp19664_c0_seq2	LG9
comp22970_c0_seq2	LG9
comp10359_c0_seq1	LG9
comp24871_c0_seq5	LG9
comp22059_c0_seq1	LG9
comp20323_c0_seq4	LG9
comp21460_c0_seq1	LG9
comp6529_c0_seq1	LG9
comp23880_c0_seq2	LG9
comp24616_c0_seq23	LG10
comp24571_c0_seq17	LG10
comp22319_c0_seq16	LG10
comp24999_c0_seq4	LG11
comp24999_c0_seq5	LG11
comp24783_c2_seq6	LG11
comp22079_c0_seq1	LG11
comp22884_c0_seq6	LG11
comp23600_c0_seq6	LG12
comp24602_c1_seq23	LG12
comp22240_c0_seq2	LG12
comp24839_c0_seq13	LG12
comp23710_c0_seq6	LG12
comp22335_c0_seq1	LG12
comp17090_c0_seq2	LG12
comp24861_c2_seq2	LG13
comp24861_c2_seq15	LG13
comp21186_c0_seq2	LG13
comp24318_c1_seq1	LG13
comp21532_c1_seq2	LG14
comp24160_c0_seq1	LG14
comp24968_c0_seq4	LG14
comp15788_c0_seq1	LG14
comp18675_c0_seq3	LG14
comp17832_c0_seq1	LG14
comp18136_c0_seq6	LG14
comp24916_c0_seq2	LG16
comp24916_c0_seq10	LG16
comp22686_c1_seq2	LG16
comp23514_c1_seq20	LG16
comp22472_c0_seq2	LG16
comp23333_c0_seq1	LG17
comp24951_c0_seq4	LG17
comp22511_c2_seq6	LG17
comp24290_c0_seq4	LG19
comp21942_c1_seq24	LG19
comp23955_c0_seq1	LG19
comp21567_c0_seq1	LG19

comp6529_c0_seq2	LG9
comp24155_c0_seq26	LG9
comp17505_c0_seq2	LG10
comp24654_c0_seq7	LG10
comp23861_c1_seq2	LG10
comp23663_c0_seq4	LG10
comp22884_c0_seq8	LG11
comp11911_c0_seq2	LG11
comp24631_c1_seq3	LG12
comp17090_c0_seq1	LG12
comp24079_c0_seq2	LG12
comp22407_c0_seq1	LG12
comp24469_c0_seq14	LG13
comp24469_c0_seq15	LG13
comp23956_c0_seq4	LG13
comp24802_c0_seq33	LG13
comp22983_c0_seq3	LG13
comp34695_c0_seq1	LG13
comp40804_c0_seq1	LG13
comp25043_c0_seq11	LG14
comp25043_c0_seq4	LG14
comp16700_c0_seq3	LG14
comp24227_c0_seq13	LG14
comp22828_c0_seq3	LG14
comp22866_c0_seq1	LG15
comp24374_c2_seq4	LG15
comp24374_c2_seq6	LG15
comp23154_c0_seq10	LG15
comp32007_c0_seq1	LG15
comp22937_c0_seq4	LG16
comp21411_c0_seq2	LG16
comp20617_c0_seq4	LG16
comp15936_c0_seq1	LG16
comp24835_c0_seq30	LG17
comp24492_c0_seq8	LG19
comp14264_c0_seq1	LG19
comp10983_c0_seq1	LG19
comp24313_c0_seq4	LG19
comp10595_c0_seq1	LG19
comp24492_c0_seq7	LG19
comp23844_c1_seq11	LG19
comp23775_c1_seq2	LG19
comp14977_c0_seq1	LG19
comp23923_c0_seq28	LG19
comp23102_c0_seq2	LG19
comp19502_c0_seq1	LG19
comp21924_c0_seq1	LG19
comp23819_c0_seq12	LG20

comp23955_c0_seq2	LG19
comp22597_c0_seq5	LG19
comp21768_c0_seq2	LG19
comp10417_c0_seq2	LG19
comp23732_c0_seq4	LG19
comp23732_c0_seq3	LG19
comp17474_c0_seq2	LG19
comp21118_c0_seq1	LG19
comp22597_c0_seq4	LG19
comp21656_c0_seq2	LG19
comp23819_c0_seq6	LG20
comp24961_c0_seq17	LG24
comp24525_c1_seq2	LG24
comp22608_c0_seq2	LG18-21
comp23298_c0_seq4	LG18-21
comp24939_c0_seq27	LG18-21
comp22608_c0_seq3	LG18-21
comp24939_c0_seq25	LG18-21
comp23032_c1_seq1	LG18-21
comp24510_c0_seq7	LG18-21
comp24883_c0_seq39	LG22-25
comp23878_c0_seq6	LG22-25
comp16962_c0_seq1	LG22-25
comp24863_c2_seq13	LG22-25
comp23267_c0_seq12	LG22-25
comp17355_c0_seq1	LG22-25
comp14496_c0_seq1	LG22-25
comp23906_c0_seq5	LG22-25
comp24023_c1_seq26	LGx
comp24823_c1_seq28	LGx

comp23819_c0_seq16	LG20
comp20329_c0_seq2	LG20
comp20898_c0_seq1	LG20
comp24586_c0_seq1	LG24
comp24019_c0_seq3	LG24
comp14053_c0_seq2	LG18-21
comp24965_c0_seq12	LG18-21
comp24965_c0_seq5	LG18-21
comp19257_c0_seq2	LG18-21
comp17406_c0_seq1	LG18-21
comp22777_c0_seq2	LG18-21
comp16893_c0_seq2	LG18-21
comp20338_c0_seq2	LG18-21
comp23298_c0_seq3	LG18-21
comp22608_c0_seq6	LG18-21
comp24883_c0_seq36	LG22-25
comp22230_c0_seq2	LG22-25
comp24762_c0_seq4	LG22-25
comp24883_c0_seq18	LG22-25
comp22230_c0_seq3	LG22-25
comp24236_c0_seq15	LG22-25
comp17766_c1_seq1	LG22-25
comp11316_c0_seq1	LG22-25
comp23372_c0_seq4	LG22-25
comp15469_c0_seq2	LG22-25
comp18913_c0_seq2	LG22-25
comp27080_c0_seq1	LG22-25
comp24587_c0_seq5	LGx
comp24581_c5_seq19	LGx
